# Occupational exposure to sharps injury among healthcare providers in Ethiopia regional hospitals

**DOI:** 10.1186/s40557-017-0163-2

**Published:** 2017-03-23

**Authors:** Nigussie Tadesse Sharew, Getaneh Baye Mulu, Tesfa Dejenie Habtewold, Kefyalew Dagne Gizachew

**Affiliations:** 1 Department of Nursing, Debre Berhan University, Debre Berhan, 445 Ethiopia; 2 Department of Epidemiology and Rob Giel Research Center, University of Groningen, Groningen, The Netherlands

**Keywords:** Sharps injury, Healthcare providers, Prevalence, Regional Hospital

## Abstract

**Background:**

Sharps injury is a penetrating stab wound from a needle, scalpel, or another sharp object that may result in exposure to blood or other body fluids. According to World Health Organization pooled estimate, the annual incidence of sharps injury in Africa was ranged from 2.10 to 4.68 per person per year, but research data in Ethiopia is limited. The aim of the study was to investigate sharps injury prevalence and associated risk factors.

**Methods:**

Institution based cross-sectional study was conducted with 200 healthcare providers (HCP) in Northeast Ethiopia. Proportionate stratified sampling was used to select HCP. Sharps injury during the last 12 months was an outcome variable whereas demographic characteristics, behavioral attributes, and job environment characteristics were independent variables. Data was collected from April to May 2016 using self-administered questionnaire; which was adapted from World Health Organization best practices for injections and related procedures toolkit. Bivariate and multivariate logistic regression analysis was carried out to identify sharps injury associated risk factors. Epi Info version 3.5.1 software package was used for data coding and entry whereas Statistical Package for Social Sciences (SPSS) version 20 software package was used for analysis.

**Results:**

In total, 195 HCP participated with a response rate of 97.5%. The prevalence of sharps injury was 32.8%. Following adjustment for covariates, lack of in-service job training and previous exposure to sharps injury were statistically significant risk factors for sharps injury. HCP who had no in-service job training were 4.7 times more likely sustained sharps injury compared with those who had in-service job training (*p* < 0.001, OR = 4.7, 95% CI = 2.05–10.56). HCP who had previous exposure to sharps injury were 3.7 times more likely sustained sharps injury compared with those who were not exposed (*p*-value = 0.002, OR = 3.7, 95% CI = 1.62–8.27).

**Conclusions:**

This study revealed 32.8% or at least three out of ten HCP exposed to sharps injury. This was found statistically significant among HCP who had no in-service job training and who had previous exposure to sharps injury. Thus, training HCP perhaps increase their skill and curiosity to reduce exposure to sharps injury.

## Background

Sharps injury is a penetrating stab wound from a needle, scalpel, or another sharp object that may result in exposure to blood or other body fluids [1]. Infectious diseases potentially transmitted by sharps injury are constantly widespread and a significant cause of illness and death [2–4]. World Health Organization (WHO) global estimate showed that every unsafe injection and needle stick injury cause at least 8 to 12 million hepatitis B infections, 2.3 to 4.7 million hepatitis C infections and 160,000 HIV/AIDS infections [4, 5]. The Center for Disease Control and Prevention (CDC) estimated that each year 385,000 sharp injury was sustained by hospital-based health care personnel [6] during the course of their duty [7]. According to WHO pooled estimate, the annual incidence of sharps injury in Africa (Egypt, Senegal, and Mauritius) was ranged from 2.10 to 4.68 per person per year [8]. Specifically, the prevalence of sharps injury was 38% in UK [9] and 19% in Kenya [10]. In addition, a cross-sectional study in Portuguese [11], South Korea [12], and Thailand [13] hospital found out the prevalence rate of sharps injury was 64.5%, 70.5% and 55.5% sharps injury respectively. Even though researchers argued decrement of sharps injury in Ethiopian hospitals [14], the incidence of sharps injury have been alarmingly increasing [15]. A cross-sectional study conducted in Bale [16] and North Shoa [17] zone, Ethiopia revealed that the prevalence of sharps injury was 19.1% and 31.5% respectively. The risk of sharps injury at the workplace was related to syringe recapping (56%), intramuscular or subcutaneous injection (22%), specimen collection or intravenous cannulation (20%), transfusion (35.5%), and inadequate waste disposal (74.8%) [1, 7, 18]. Furthermore, other studies identified suturing, removing the needle from syringes after injection, sharps disposal were risky procedures that expose to sharps injury [13, 19]. There were several factors associated with increased risk of sharps injury: lack of training, extended working hours, job dissatisfaction, work experience, and perception of risk [20]. Moreover, age, poor compliance with infection-control procedures, and inadequate knowledge of blood-borne pathogens were associated factors for sharps injury [21].

The persistence of preventable, life-threatening occupational hazard particularly sharps injury at the hospital is yet to be given attention in developing countries including Ethiopia [8]. Therefore, the aim of the study was to investigate sharps injury prevalence and associated risk factors.

## Methods

### Study setting

The study was conducted in Debre Berhan Town, Northeastern Ethiopia. Debre Berhan is the capital city of North Shoa zone and located 130 km North East from Addis Ababa, the capital city of Ethiopia. The city has nine kebeles with a total population of 94,829 (50.8% female). There are 22 health institutions: two hospitals, three health centers, and 17 private clinics [17]. The study was conducted at the two hospitals considering the magnitude of sharps injury is high.

### Study design and population

Institution based cross-sectional study was conducted from April to May 2016. The total number of source population in the two Hospitals was 434 healthcare providers (HCP): 384 at Debre Berhan Referral Hospital and 50 at Ayu General Hospital. All HCP who were actively involved in the patient care, fully employed, and at least with one-year work experience were included. However, HCP and managers who were on holiday, sick leave, and maternal leave were excluded.

### Sample size and sampling procedure

The sample size was calculated using single population proportion formula. Given the prevalence of sharps injury was 28% [22], 95% confidence level, 5% marginal error, and 10% non-response rate, the final sample size was 200. Systematic random sampling (sampling interval /k/=2) method was used for selecting HCP.

### Study variables

Sharps injury during the last 12 months was an outcome variable. The explanatory variables were demographic characteristics, behavioral attributes, and job environment characteristics. ‘Previous exposure to sharps injury’ was defined as observing the incident of sustained sharps injury by someone or inflicting sharps injury to oneself during the last 12 months. Besides, ‘previous exposure of needle recapping’ was defined as observing someone recapping a needle or needle recapping by oneself during the last 12 months.

### Data collection

Self-administered structured questionnaire, which was adapted from WHO best practices for injections and related procedures toolkit [23], was used for data collection. The questionnaire has three sections: section 1_demographic characteristics; section 2_sharps injury; and section 3_behavioral attributes and job environment characteristics. First, the questionnaire was developed in English. Next, it was translated using the forward-backward method from English to Amharic (local language) by professional fluent in both languages. Finally, it was pre-tested at Kebele 04 health center. Five nursing intern students collected the data.

### Data analysis

Before data coding and entry, the supervisor and investigators reviewed and checked each questionnaire for completeness, accuracy, and consistency. Printed frequency was used for checking missingness and outlying values. To test the hypothesized association of each explanatory variable with the outcome variable, bivariate and multivariate logistic regression analysis was done. Variables reached a *p*-value ≤0.25 were included in the final model. Variables with *p*-value ≤0.05 in the final full model test were identified as independently associated risk factors. Odds ratio with 95% confidence interval was used to measure the strength of association. Numeric summary measures, tables, and figures were used to present the data. The study was adherent to the Strengthening the Reporting of Observational Studies in Epidemiology (STROBE) statement [24]. Epi Info version 3.5.1 software package was used for data coding and entry whereas Statistical Package for Social Sciences (SPSS) version 20 software package was used for analysis.

## Results

### Demographic characteristics

In total, 195 HCP participated with a response rate of 97.5%. More than half (56.4%) of HCP were between the age of 25 and 30 years, 59.0% were female, 50.3% were married, 90.3% were working at Debre Berhan Referral hospital, and 43.6% were Nurse (Table 1).

**Table 1:** Demographic characteristics of HCP

Variables	Categories	*n* = 195	%
Sex	Male	80	41.0
	Female	115	59.0
Marital status	Married	98	50.3
	Single	97	49.7
Age	<25	31	15.9
	25–30	110	56.4
	31–40	42	21.5
	>40	12	6.2
Experience year	1–5	109	55.9
	5–9	57	29.2
	10–14	18	9.2
	≥15	11	5.6
Profession	Nurse	85	43.6
	Midwife	21	10.8
	Medical laboratory science	23	11.8
	Others^a^	66	33.8
Name of hospital	Debre Berhan Referral Hospital	176	90.3
	Ayu hospital	19	9.7
Unit	Emergency	22	11.3
	Outpatient department	32	16.4
	Pediatrics	23	11.8
	Medical	20	10.3
	Surgical	23	11.8
	Maternal and child health	35	17.9
	Laboratory	25	12.8
	Others^b^	15	7.7
HBV vaccination	Yes	23	11.8
	No	172	88.2

^a^Physicians, Porters, Health officers, Anesthetist, Emergency Surgeons

^b^Ophthalmology, dental, psychiatry, anesthesia

### Sharps injury

The prevalence of sharps injury was 32.8%. Table 2 presented, at least one out of five (21.5%) HCP sustained sharps injury once during the last 12 months. The most abundant type of sharps injury was needle stick injury (21.4%) followed by intravenous cannula (5.6%). Sudden movement of patients (20.5%) was the most frequent mechanism of sharps injury.

**Table 2:** Sharps injury among HCP

Variables	Categories	*n* = 64	%
Incident of sharps injury during the last 12 months	One times	42	21.5
	Two times	18	9.2
	Three times	3	1.5
Incident of sharps injury during the last month	One times	19	9.7
	Not injured	45	23.1
Type of sharps injury	Needle sticks	42	21.4
	Glasses item	4	2.1
	Intravenous cannula	11	5.6
	Scalpel blade	4	2.1
	Others	3	1.5
Type of sharps injury	Slight skin penetration	15	7.7
	Superficial	46	23.6
Mechanism of sharps injury	During recapping	8	4.1
	Sudden movement of patients	40	20.5
	During sharp collection	11	5.6
	Other	5	2.6
Part of body injured	Hand	32	16.4
	Finger	31	15.9
Mechanism of sharps injury inflicted	Self	60	30.8
	Non-compliant patient	4	2.1

### Behavioral attributes and job environment characteristics

Nearly three-fourth (69.7%) of HCP knew the department to report sharps injury. Half (50.3%) of HCP reported sharps material outside the sharps collection box. Seventy-five (38.5%) HCP reported the sharps collection box was available at distance of hand stretch (Table 3).

**Table 3:** Behavioral attributes and job environment characteristics

Variables	Categories	*n* = 195	%
Behavioral attributes			
Previous exposure of needle recapping	yes	74	37.9
	no	121	62.1
Previous exposure to sharps injury	yes	64	32.8
	no	131	67.2
Awareness of the department to report sharps injury	yes	136	69.7
	no	59	30.3
In-service job training	yes	92	47.2
	no	103	52.8
Job environment			
Infection prevention committee	Yes	130	66.7
	No	65	33.3
Source of syringe with needle	Patient bought from health facility	167	85.6
	Free of charge from facility	28	14.4
Post-exposure management in the facility	yes	141	72.3
	no	54	27.7
Privacy during counseling	yes	134	68.7
	no	61	31.3
Injection environment	clean	185	94.9
	dirty and contaminated	10	5.1
Availability of sharp materials collection box	yes	156	80.0
	no	39	20.0
Status of sharp materials collection box	over-filled	51	26.2
	3/4^th^	144	73.8
Sharp materials outside the collection box	yes	98	50.3
	no	97	49.7
Way of disposal of sharp materials	open incineration	41	21.0
	protected incineration	109	55.9
	open dumping	32	16.4
	burial in a pit	13	6.7
Availability of guideline	yes	94	48.2
	no	101	51.8
Emergency care procedure	yes	28	14.4
	no	167	85.6
Injection procedure	yes	11	5.6
	no	184	94.4
Suturing procedure	yes	11	5.6
	no	184	94.4
Sharps collection box availability at distance of hand stretch	yes	120	61.5
	no	75	38.5

### Association between demographic characteristics and sharps injury

As shown in Table 4, the profession was found significant risk factor for sharps injury. Midwifery professionals were 2.8 times more likely exposed to sharps injury compared with physicians, health officers, emergency surgeons, and anesthetist (*p*-value = 0.04, OR = 2.8, 95% CI = 1.02–7.92).

**Table 4:** Bivariate association of demographic characteristics and sharps injury

Variables (reference category)		*n* = 195	%	*p*-value	OR (95% CI)
Sex (female)		80	41.0	0.40	1.3 (0.71, 2.38)
Marital status (single)		98	50.3	0.24	1.4 (0.78, 2.61)
Ayu hospital (Debre Berhan Referral Hospital)		19	9.7	0.16	2.0 (0.76, 5.15)
No hospital infection prevention committee		65	33.3	0.39	1.3 (0.70, 2.46)
HBV vaccinated		23	11.8	0.50	1.4 (0.56, 3.35)
Age (year) (>40 years)	<25	31	15.9	0.24	2.8 (0.51, 14.86)
	25–30	110	56.4	0.24	2.5 (0.53, 12.17)
	31–40	42	21.5	0.28	2.5 (0.48, 12.99)
Experience (year) (≥15 years)	<5	109	55.9	0.22	2.7 (0.56, 13.18)
	5–9	57	29.2	0.50	1.7 (0.34, 9.03)
	10–14	18	9.2	0.56	1.7 (0.27, 10.97)
Profession (Others^a^)	Nurse	85	43.6	0.14	1.7 (0.83, 3.49)
	Midwife	21	10.8	0.04	2.8 (1.02, 7.92)
	Laboratory	23	11.8	0.33	1.7 (0.60, 4.65)
Unit (Others^b^)	Emergency	22	11.3	0.38	1.9 (0.46, 7.92)
	Outpatient department	32	16.4	0.54	0.6 (0.15, 2.70)
	Pediatrics	23	11.8	0.60	1.5 (0.35, 6.13)
	Medical ward	20	10.3	0.60	1.5 (0.34, 6.43)
	Surgical ward	23	11.8	0.60	1.5 (0.35, 6.13)
	Maternal and child health	35	17.9	0.48	1.6 (0.43, 6.17)
	Laboratory	25	12.8	0.54	1.5 (0.38, 6.31)

^a^Physicians, Porters, Health officers, Anesthetist, Emergency Surgeons

^b^Ophthalmology, dental, psychiatry, anesthesia

*OR* Odds ratio

*CI* Confidence interval

### Association between behavioral attributes and job environment characteristics and sharps injury

As presented in Table 5, lack of in-service job training (*p* < 0.001, OR = 6.0, 95% CI = 2.95–12.03), previous exposure to sharps injury (*p* < 0.001, OR = 3.4, 95% CI = 1.82–6.48), unavailability of universal precaution guideline (*p*-value = 0.02, OR = 2.1, 95% CI = 1.14–3.90), and unavailability of sharps collection box at distance of hand stretch (*p*-value = 0.02, OR = 2.1, 95% CI = 1.11–3.77) were statistically significant associated risk factors for sharps injury.

**Table 5:** Bivariate association of behavioral attributes and job environment characteristics and sharps injury

Variables	*n* = 195	%	*p*-value	OR (95% CI)
Syringe with needle used free of charge from hospital	28	14.4	0.23	1.7 (0.73, 3.76)
No post-exposure management at the hospital	54	27.7	0.93	1.0 (0.53, 2.01)
No awareness on department to report sharps injury	59	30.3	0.38	1.3 (0.70, 2.53)
No privacy during counseling	61	31.3	0.33	1.4 (0.73, 2.59)
Lack of in-service job training	103	52.8	<0.001	6.0 (2.95, 12.03)
Previous exposure of needle recapping (self or others)	74	37.9	0.14	1.6 (0.68, 2.92)
Previous exposure to sharps injury (self or others)	64	32.8	<0.001	3.4 (1.82, 6.48)
Dirty and contaminated injection environment	10	5.1	0.24	2.1 (0.59, 7.66)
Unavailability of sharps materials collection box	39	20.0	0.94	1.0 (0.49, 2.17)
Status of sharp materials collection box	144	73.8	0.80	1.1 (0.55, 2.17)
Previous exposure to sharps materials outside the collection box	98	50.3	0.08	1.7 (0.94, 3.17)
Open dumping disposal of sharps materials	32	16.4	0.08	0.4 (0.11, 1.13)
Unavailability of universal precaution guideline	101	51.8	0.02	2.1 (1.14, 3.90)
Unavailability sharps collection box at distance of hand stretch	75	38.5	0.02	2.1 (1.11, 3.77)

*OR* Odds ratio

*CI* Confidence interval

Following adjustment for covariates, lack of in-service job training and previous exposure to sharps injury were statistically significant risk factors for sharps injury. HCP who had no in-service job training were 4.7 times more likely sustained sharps injury compared with those who had in-service job training (*p* < 0.001, OR = 4.7, 95% CI = 2.05–10.56). HCP who had previous exposure to sharps injury were 3.7 times more likely sustained sharps injury compared with those who were not exposed (*p*-value = 0.002, OR = 3.7, 95% CI = 1.62–8.27) (Table 6).

**Table 6:** Multivariate analysis of selected variables and sharps injury

Variables	*n* = 195	%	*p*-value	OR (95% CI)
Marital status, married	98	50.3	0.22	1.7 (0.74, 3.67)
Age,31–40 years	42	21.5	0.10	5.4 (0.72, 40.04)
Work experience, <5 years	109	55.9	0.30	3.0 (0.38, 23.50)
Profession, Midwifery	21	10.8	0.76	1.3 (0.29, 5.52)
Hospital, Ayu general hospital	19	9.7	0.56	1.4 (0.43, 4.76)
Syringe with needle used free of charge from hospital	28	14.4	0.70	1.3 (0.39, 4.09)
Lack of in-service training	103	52.8	<0.001	4.7 (2.05, 10.56)
Previous exposure of needle recapping (self or others)	74	37.9	0.83	1.1 (0.50, 2.44)
Previous exposure to needle stick injury (self or others)	64	32.8	0.002	3.7 (1.62, 8.27)
Dirty and contaminated injection environment	10	5.1	0.27	2.5 (0.50, 12.51)
Previous exposure to sharp materials outside the collection box	98	50.3	0.58	1.2 (0.58, 2.68)
Open dumping disposal of sharps materials	32	16.4	0.81	1.2 (0.25, 5.91)
Unavailability of universal precaution guideline	101	51.8	0.24	1.6 (0.72, 3.67)
Unavailability sharps collection box at distance of hand stretch	75	38.5	0.50	1.3 (0.58, 3.09)

*OR* Odds ratio

*CI* Confidence interval

## Discussion

At least twenty large occupational groups are exposed to biohazards [25]. The risk is greatest among healthcare and laboratory workers who are threatened by the transmission of human pathogens including Hepatitis B Virus (HBV), Hepatitis C Virus (HCV), HIV/AIDS virus, malaria, syphilis, tuberculosis, brucellosis, herpes virus and diphtheria [7]. However, the World Health Organization (WHO) estimated 25–90% exposure to sharps injury remain unreported [7]. This study aimed to uncover the prevalence and associated risk factors of sharps injury among hospital HCP.

The prevalence of sharps injury among HCP during the last 12 months was 32.8%, which was in agreement with the prevalence rate reported in Ethiopia [17, 20] and United Kingdom [9]. However, the prevalence rate in our study was higher than the study report by Mbaisi et al. [10] in Kenya, Gessessew and Kahsu [26] and Bekele et al. [16] in Ethiopia. This inconsistency may be due to the difference in the operationalization of sharps injury, study population, the number of HCP in the facility, different work environment and culture and availability of resources [20]. In this study, the prevalence of sharps injury was highest in Midwifery professionals compared with nurses, physicians, health officers, emergency surgeons, and anesthetist. However, several earlier studies [16, 20] concluded sharps injury was highest in nurses.

In this study, the commonest mechanism of sharps injury was self-inflicted needle stick injury due to sudden movement of patients. Contrariwise, the Pakistani study indicated that the most commonly reported mechanisms for sharps injury were injection and two-handed recapping of the needle [19]. To avoid sudden movement, it is believed patients should be informed prior to any procedures that cause pain to prepare them psychologically. Agitated patients should be restrained manually or relevant medication should be given to calm them.

In accordance with earlier finding [20], multivariate logistic regression analysis in this study showed that a lack of in-service job training and previous exposure to sharps injury were significant risk factors for sharps injury. Due to lack of training, HCP may not have sufficient knowledge and skill to prevent sharps injury and perhaps increased the risk of injury as a result. A study carried out in Sub-Saharan Africa also supported the importance of training among HCP [27]. Previous exposure to sharps injury may decreased HCP perceived risk of sharps injury and they did not take precaution. This was supported by a study done in Gonder, Ethiopia where HCP with low perceived risk of sharps injury might not take special care to avoid injury while performing different activities using sharp materials [20].

Opposite to the conclusion by Bekele et al. [16], our study revealed that there was no statistically significant association between HCP work experience and rate of sharps injury. Our finding also supported by a study conducted in Northern Ethiopia [20] where work experience did not affect the risk of sharps injury. HCP working in the emergency unit had a higher risk of sharps injury compared with ophthalmology, dental, psychiatry, anesthesia unit workers. The possible explanation was that critical and risky procedure executed in the emergency unit [16].

The strengths of this study include a high response rate and the inclusive nature of this research as HCP could participate from all profession. Including hospitals where risky procedures carried out was a further strength. Moreover, a reasonable sample size was used. However, this study had certain limitations. Since the study was self-administered and the last 12 months incident was evaluated, social desirability and recall bias might added. Due to cross-sectional nature of the study, only temporal association was assumed between sharps injury and identified risk factors.

## Conclusions

This study revealed 32.8% or at least three out of ten HCP exposed to sharps injury. This was found significant among HCP who had no in-service job training and who had previous exposure to sharps injury. Thus, the training of HCP should always be undertaken for new employees and periodically for those already employed. Moreover, periodical assessment of HCP knowledge and skills and training about the use of new medical equipments helps to prevent exposure to sharps injury [7].
